# Changes in pain sensitivity and spinal stiffness in relation to responder status following spinal manipulative therapy in chronic low Back pain: a secondary explorative analysis of a randomized trial

**DOI:** 10.1186/s12891-020-03873-3

**Published:** 2021-01-06

**Authors:** Casper Glissmann Nim, Gregory Neil Kawchuk, Berit Schiøttz-Christensen, Søren O’Neill

**Affiliations:** 1 Medical Research Unit, Spine Center of Southern Denmark, University Hospital of Southern Denmark, Østrehougvej 55, 5500 Middelfart, Denmark; 2 Department of Regional Health Research, University of Southern Denmark, Campusvej 55, 5230 Odense M, Denmark; 3 Department of Physical Therapy, University of Alberta, 8205 114St, 2-50 Corbett Hall, Edmonton, Alberta T6G 2G4 Canada

**Keywords:** Spinal manipulative therapy, Lumbar stiffness, Pain sensitivity, Responder analysis

## Abstract

**Background:**

In a prior randomized trial, we demonstrated that participants receiving spinal manipulative therapy at a pain-sensitive segment instead of a stiff segment experienced increased mechanical pressure pain thresholds. We hypothesized that the targeted segment mediated this increase through a segment-dependent neurophysiological reflective pathway. Presently, it is not known if this decrease in pain sensitivity is associated with clinical improvement. Therefore, we performed an explorative analysis to examine if changes in experimental pain sensitivity (mechanical and thermal) and lumbar stiffness were further dependent on clinical improvement in disability and patient-reported low back pain.

**Methods:**

This study is a secondary explorative analysis of data from the randomized trial that compared 132 participants with chronic low back pain who received lumbar spinal manipulative therapy applied at either i) the stiffest segment or ii) the segment having the lowest pain threshold (i.e., the most pain-sensitive segment). We collected data at baseline, after the fourth session of spinal manipulation, and at 14-days follow-up. Participants were dichotomized into responders/non-responders using different clinical variables (disability and patient-reported low back pain) with varying threshold values (0, 30, and 50% improvement). Mixed models were used to assess changes in experimental outcomes (stiffness and pain sensitivity). The fixed interaction terms were time, segment allocation, and responder status.

**Results:**

We observed a significant increase in mechanical pressure pain thresholds for the group, which received spinal manipulative therapy at the most pain-sensitive segment independent of whether they improved clinically or not. Those who received spinal manipulation at the stiffest segment also demonstrated increased mechanical pain sensitivity, but only in the subgroup with clinical improvement. We did not observe any changes in lumbar stiffness.

**Conclusion:**

Our results suggest the existence of two different mechanistic pathways associated with the spinal manipulation target. i) A decrease of mechanical pain sensitivity independent of clinical outcome (neurophysiological) and ii) a decrease as a reflection of the clinical outcome. Together, these observations may provide a novel framework that improves our understanding of why some respond to spinal manipulative therapy while others do not.

**Trial registration:**

ClinicalTrials.gov identifier: NCT04086667 registered retrospectively September 11th 2019.

**Supplementary Information:**

The online version contains supplementary material available at 10.1186/s12891-020-03873-3.

## Background

### Spinal manipulative therapy

Clinical guidelines recommend spinal manipulative therapy (SMT) for chronic low back pain (LBP) [1], given its effect on pain and disability is comparable to that of other recommended conservative therapies [2]. Toward optimizing SMT for chronic LBP, we recently published a randomized clinical trial (RCT) [3]. In which, we experimentally categorized each segment as having either high stiffness or high mechanical pain sensitivity. In this trial, the participants received four SMT sessions directed at either the segment with the highest stiffness or highest pain sensitivity in a randomized manner. While significant within-group changes emerged in patient-reported LBP, the randomization did not yield any between-group differences. We also re-measured the experimental outcomes (stiffness and mechanical pain sensitivity) at each time point in the course of treatment. While we did not find differences in lumbar stiffness, mechanical pain sensitivity decreased significantly in the group that received SMT at segments characterized by high mechanical pain sensitivity (*pain segment group*), compared to the group that received treatment at segments characterized by high stiffness (*stiff segment group*). Interestingly, this increase did not imply clinical pain reduction. On this basis, we hypothesized that a segment-specific neurophysiological reflex most likely mediated the observed effect on mechanical pain sensitivity as opposed to a curative effect on a mechanical dysfunction [3].

### Impact of being a responder

Our prior paper’s analysis did not investigate the potential impact of being a clinical responder or non-responder within the allocated subgroups. For instance, preliminary research suggests decreases in lumbar stiffness following SMT, but only when this corresponded with improvements in disability [4, 5]. A similar pattern may emerge in this cohort when applying a responder threshold.

Notably, the finding that a segmental effect on mechanical pain sensitivity is not a proxy for clinical improvement appears inconsistent with previous studies that have found a hypoalgesic effect after overall clinical improvement [6, 7]. However, interpreting our novel finding of a modulating segmental impact of SMT requires a thorough responder analysis. Possible within-group differences in mechanical pain sensitivity could have affected our prior results. Besides, our previous study only included mechanical deep pressure pain sensitivity. Including other types of stimuli associated with chronic LBP [8], like peripheral heat pain sensitivity, may be helpful in an explorative and secondary analysis, as would be the impact of segmental versus regional measurements.

### Objectives

In this explorative analysis, we used patient-reported disability and low back pain intensity to determine responder status and examine its relationship to various experimental outcome measures (changes in lumbar stiffness, mechanical pain sensitivity, and heat pain sensitivity).

## Methods

### Setting

A pre-planned secondary explorative analysis of data used for a primary analysis in an RCT (registered at Clinical.Trial.gov identifier: NCT04086667) [3]. One-hundred-and-thirty-two participants enrolled in the study. All participants received lumbar spinal manipulative therapy directed randomly at either the segment of highest stiffness or the segment of highest mechanical pain sensitivity. Seven participants did not complete the study. Two others were lost to follow-up, leaving 123 participants with complete data sets.

We included participants from a regional Spine Center in Denmark. The inclusion criteria were 18 to 60-year-old patients with LBP of benign or degenerative origin for more than three months. No prior spinal surgery and no indication for current spinal surgery. No history of SMT in the preceding four weeks. Body mass index had to be below 35. We limited the daily opioid intake to 40 mg of morphine at the time of inclusion.

Exclusion criteria were: failure to complete 75% of the allocated SMT interventions, receiving manual therapy of the lower back in another, non-project setting, or change pain medication during the study period. We excluded no patients after initiating the project based on these criteria.

All participants gave oral and written informed consent for the study approved by the regional research ethics board (S-20160201).

### Procedure

The primary study protocol is described in full detail in the prior study [3]. A short overview is provided here.

We extracted demographic data from the SpineData questionnaire, a clinical registry in use at The Spine Center of Southern Denmark [9].

The *baseline* lab session consisted of i) completing patient-reported clinical outcomes, ii) identification and marking of each spinous process for each lumbar segment using ultrasonography (Sonosite Titan Linear, L38 probe) [10], iii) measures of lumbar stiffness and pain sensitivity (mechanical and heat), iv) segment randomization (high stiffness or high mechanical pain sensitivity) and v) initial SMT application.

Three additional SMT applications were provided over the following 14 days.

After the fourth SMT application, the *post-SMT* lab session followed in which we repeated items i - iii from the baseline lab session.

A final *follow-up* lab session took place approximately 14 days after the *post-SMT* session. Again, we repeated items i - iii. This concluded the study.

#### Spinal manipulative therapy

Two chiropractors (see acknowledgments), each with more than 12 years of clinical experience, performed the SMT, both blinded to the segment target allocation. Participants received standardized SMT that consisted of a side-lying posterior to anterior high velocity, low amplitude thrust with contact point at the spinous process of the indicated segment. The protocol allowed up to three attempts for a successful treatment determined subjectively by the chiropractor and independently of the joint-related sounds that can accompany SMT [11].

### Responder variables and responder thresholds

We conducted the responder analyses with two variables using three different responder thresholds. *Disability*, assessed by the Oswestry Disability Index (ODI) (version 2.1), was used to dichotomize responder status using three different responder thresholds: i) more than or equal to a 50% improvement to better be able to compare with similar literature [4, 5, 12], ii) more than or equal to a 30% improvement as consensus recommended [13], and iii) more than or equal to a 0% improvement indicating the absolute dichotomization between worsening and improving. The ODI has been translated to Danish and validated as a reliable instrument to measure LBP changes [14].

Similarly, we used *patient-reported low back pain* to set responder status at the same three different responder thresholds: i) a 50% improvement, ii) a 30% improvement, and iii) 0% improvement. The pain intensity score used a mean score (0–10) from the self-reported LBP numerical rating scale (NRS) [15], which consists of three scales: Current LBP, Worst LBP in the previous 14 days, and Average LBP in the last 14 days.

We only performed each dichotomization at the final follow-up time point.

### Experimental measures

#### Lumbar stiffness

Lumbar stiffness was measured using the custom-manufactured research tool *VerteTrack* (VT). The apparatus consists of a pair of rollers, loaded by a fixed weight, which moves along the lumbar spine with one wheel on either side of the midline (3 cm apart). The movement is controlled in two axes (superior/inferior and medial/lateral) by computer-controlled stepper motors. Thus, reliably tracking a specific and pre-determined path (skin markings) along the spine. Displacement in the third axis (anterior/posterior) is measured continuously during movement by a string potentiometer (TE Connectivity, USA). Therefore, the VT generates a series of vertical displacement data for a given fixed weight in relation to the longitudinal and transverse positions. In the current study, VT measurements were performed with increasing weights, from 0 to 6 kg in steps of 1 kg. The VT has a sampling rate of 30 Hz. The participants were in a prone position during the procedure, and each indentation took approximately 8 s. Participants were guided to exhale and hold their breath while the rolling of the wheels transpired.

The VT has primarily been validated as safe and reliable in healthy volunteers [16, 17]. No study has yet examined the validity when applied to a back pain population. However, this has been achieved in a prior version using single indentation instead of rolling indentation [18]. The bench-top performance indicates that the VT is accurate in-vivo both for rolling and single indentation [19]. Thus, suggesting that the use of the VT was feasible in this cohort.

We measured stiffness for each segment as a global stiffness (GS) score, which denotes the force-displacement curve’s average slope from the second load to the second heaviest load tolerated. Thus, GS was available i) for each segment (e.g., L5), and ii) each segment’s GS score was averaged as a single score indicating the GS score for the entire lumbar spine (L1 to L5).

#### Mechanical pain sensitivity

We assessed pressure pain threshold (PPT) using a pressure algometer (Somedic Model 2, Sweden). Attached to the probe was a custom, 3D printed double-headed probe (2 × 1 cm^2^, 3 cm apart), which allowed for a bilateral pressure to be applied at either side of the midline corresponding to the point of indentation for the VT. The rate of increase in pressure was kept at a near-constant 50 kPa/s (indicator on the algometer). Each segment was measured three times in random order with 10-s rest intervals. The participant indicated when the pressure was perceived as painful by pressing an indicator button. We recorded this score as the PPT. If no pain had been elicited by 1000 kPa, this was recorded as the PPT. If the first and second measurements on a given segment were 1000 kPa, we did not perform a third. Pressure pain threshold has previously been shown to have excellent intra-rater reliability in a back pain population [20].

We averaged the PPT score (kPa) from each of the three trials for i) each segment (e.g., L5), and then for ii) all segments (i.e., L1 to L5).

#### Heat pain sensitivity

Heat pain threshold (HPT) was measured shortly after PPT. The thermode (Medoc TSA-II, Israel) used a single 3x3cm probe applied to the midline. The thermode baseline temperature was pre-set to 32 degrees Celsius. It was increased at a rate of 1 degree per second during testing until the participants indicated that the stimulation was perceived as painful by pressing an indicator button connected to the thermode controller. When the participant indicated the stimulation as painful, the probe was lifted off the skin without delay, and the temperature returned to the baseline temperature (10 degrees/second). Each segment was measured three times in random order with 10-s rest intervals. If no pain had been indicated at 50 degrees, this was recorded as the HPT, and the thermode returned automatically to baseline temperature. When applied to the spine, HPT has previously been found to have good-to-excellent intra-rater reliability in a healthy population [21].

We averaged the HPT score (C) from each of the three trials for i) each segment (e.g., L5), and then for ii) all segments (i.e., L1 to L5).

Before data collection, a trial run consisting of 1–2 tests on the lower extremity and at the T12 vertebra was performed for both PPT and HPT to familiarize the participant with the procedures.

### Segmental randomization

The randomization process is described in full detail in the primary study [3]. In short, segmental stiffness was determined using the raw force-displacement data (mm) from the VT’s heaviest individual load (typically 6 kg), and segmental mechanical pain sensitivity was determined as the mean value of the three PPT measures. To resolve situations where the segments with the highest pain sensitivity and stiffness were identical or adjacent, a ratio ranging between − 1 and + 1 was calculated for each segment based on these two variables in combination. A ratio approximating − 1 would indicate high stiffness and low pain sensitivity, and correspondingly a ratio approaching + 1 would indicate high pain sensitivity and low stiffness. SMT was directed at the segment with the highest (or lowest) ratio index.

### Statistical analysis

Frequencies (count) are presented for responders/non-responders as defined by each threshold, and the cumulative proportion of responders is presented graphically for both ODI and NRS.

We performed a three-way mixed model analysis with subject as a random intercept using an unstructured variance-covariance structure. The interacting fixed effects were segment-allocation (target site), time, and responder status to determine within-group changes and between-group differences. For a concise description of the mixed model approach, see Bates et al. [22]. Responder status consisted of six different predictor variables (minimum or equal to 50, 30, and 0% change in both ODI and NRS). The mixed model assumptions were upheld for the models. They evaluated: i) normal distribution of the residuals error using Q-Q plots, and ii) the homogeneity of variance by visually inspecting the residuals versus the predicted values.

We had to omit approximately 11% of the data points due to inaccuracies when identifying the segments at different time points. See the primary study for further detail [3].

The mixed models are presented as mean changes within-group from baseline to post-SMT and follow-up along with 95% confidence intervals. Where significant within-group changes are present in any outcomes, a table is present to further describe between-group mean differences (responder versus non-responder).

#### Segmental proximity analysis

In addition to the mixed model described above, we also categorized the lumbar segments into three groups: i) the specific SMT targeted segment (e.g., *L2*), ii) the adjacent segment(s) to the targeted (e.g., *L1* and *L3*), and iii) other segments (e.g., *L4* and *L5*). This segmental categorization was added as an interaction term to the original three-way models for the experimental outcomes (GS, PPT, and HPT) for ODI and NRS.

We completed the data analyses in R [23] (Linux, v. 3.6.0 with R-studio v. 1.1.456). Data wrangling was completed using the *Tidyverse* [24]. The mixed models were fitted using the *lme4* package [22], *p*-values for the mixed models were calculated using the *multcomp* package [25]. A p-value < 0.05 was considered significant, and repeated p-values were adjusted using the single-step method [25].

## Results

### Proportion of responders

Table 1 present the responder/non-responder distribution of the 123 participants who completed the intervention.

**Table 1:** Proportion of responders/non-responders for ODI and NRS at all responder thresholds (0, 30 and 50%)

Parameter	≥0% improvement n(%)	≥30% improvement n(%)	≥50% improvement n(%)
ODI Responder	95 (77)	46 (37)	29 (24)
ODI Non-Responder	28 (23)	77 (63)	94 (76)
NRS Responder	94 (76)	35 (28)	19 (15)
NRS Non-Responder	29 (24)	88 (72)	104 (85)

*ODI* Oswestry disability index, *NRS* self-reported LBP numerical rating scale

**Fig. 1:**
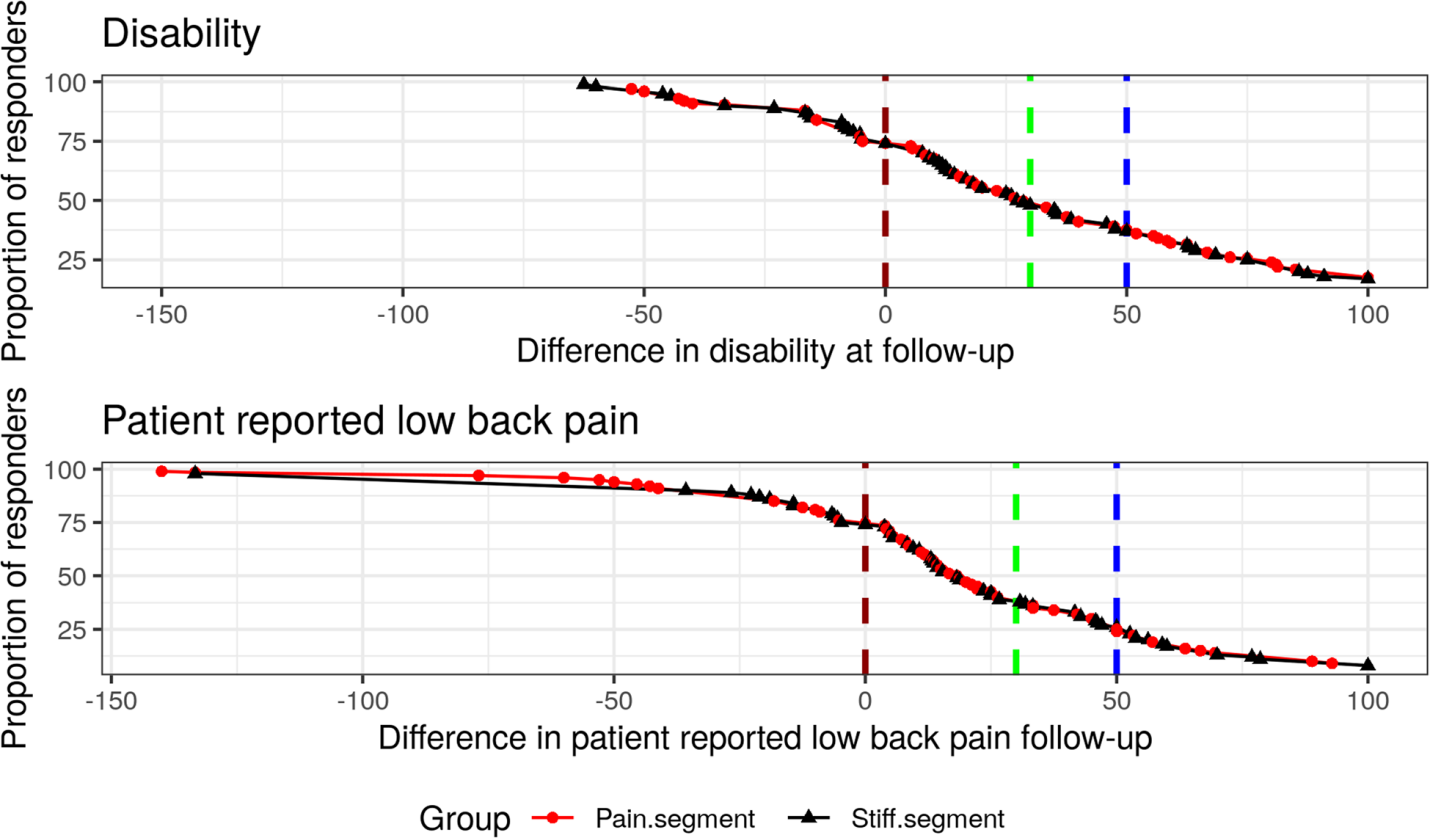
Cumulative proportion of responders. A cumulative responder proportion graph for the participants treated with spinal manipulative therapy at either a *pain segment* or a *stiff segment*. The red line indicates a 0% improvement, the green line indicates a 30% improvement, and the blue line indicates a 50% improvement. Improvements are shown for disability and patient-reported low back pain. A negative value indicates worsening of the outcomes

Figure 1 illustrates the cumulative proportion of responders for ODI and NRS at follow-up. The vertical dashed lines represent thresholds for responder/non-responder at 50% (blue), 30% (green), and 0% (red). The line extends negatively beyond 0, indicating that 28 (23%) of the cohort experienced worsening of ODI at follow-up, and 29 (24%) demonstrated worsening of NRS. It also shows that the two groups respond at an equal rate. Furthermore, while an equal number of participants has increased (worsening) ODI and NRS, the variance is much greater in NRS changes, approximating 150% versus 50% for ODI.

### Responder analysis

We present the results of the responder analysis in Tables 2 and 3. Table 2 lists changes in objective outcome measures (GS, PPT, and HPT) immediately post-SMT (4th session) and at follow-up for the two randomization groups (SMT at the stiffest segment and most pain-sensitive segment) and subgrouped by ODI responder status defined as 0, 30, and 50% improvement thresholds. Table 3 presents similar data for NRS.

**Table 2:** Changes for responders (ODI) in lumbar stiffness and pain sensitivity (mechanical and thermal) following SMT

Time	Status	Threshold	GS		PPT		HPT	
			Pain	Stiff	Pain	Stiff	Pain	Stiff
Post-SMT	Responder	50%	− 0.20(− 0.70:0.31)	− 0.13(− 0.77:0.52)	110 (19:200)*	94(− 24:211)	0.6(− 0.9:2.0)	1.2(− 0.7:3.1)
		30%	− 0.11(− 0.53:0.32)	− 0.13(− 0.61:0.34)	103 (27:178)*	109 (24:195)*	0.2(− 1.1:1.4)	1.1(− 0.3:2.5)
		0%	−0.01(− 0.31:0.28)	−0.06(− 0.38:0.26)	92 (39:145)*	62 (4:120)*	0.2(− 0.7:1.1)	0.5(− 0.5:1.4)
	Non-Responder	50%	0.10(−0.23:0.42)	0.06(−0.25:0.37)	95 (37:153)*	19(− 36:75)	− 0.2(− 1.1:0.8)	0.1(− 0.8:1.0)
		30%	0.10(− 0.26:0.45)	0.11(− 0.24:0.45)	96 (34:159)*	−6(−67:55)	− 0.1(− 1.1:0.9)	−0.1(− 1.1:0.9)
		0%	0.16(−0.48:0.80)	0.26(−0.26:0.79)	135 (17:253)*	− 45(− 141:50)	− 0.7(− 2.6:1.2)	0.0(− 1.6:1.5)
Follow-up	Responder	50%	− 0.22(− 0.74:0.30)	0.12(− 0.50:0.74)	143 (48:237)*	91(−22:204)	1.3(− 0.2:2.8)	1.1(− 0.9:3.1)
		30%	0.04(− 0.40:0.47)	0.07(− 0.41:0.55)	105 (28:182)*	125 (40:211)*	1.1(− 0.2:2.4)	0.5(− 1.0:2.0)
		0%	0.20 (0–0.10:0.51)	− 0.07(− 0.40:0.26)	87 (32:142)*	75 (15:135)*	0.9(− 0.0:1.8)	0.8(− 0.2:1.8)
	Non-Responder	50%	0.19(−0.14:0.51)	− 0.10(− 0.42:0.22)	70 (11:128)*	38(− 21:96)	0.3(− 0.7:1.3)	0.7(− 0.3:1.6)
		30%	0.10(− 0.26:0.46)	−0.12(− 0.48:0.24)	79 (15:143)*	6(− 57:70)	0.2(− 0.9:1.3)	0.9(− 0.2:2.0)
		0%	− 0.44(− 1.06:0.18)	0.00(− 0.54:0.54)	103(− 10:217)	− 23(− 121:75)	−0.6(− 2.5:1.2)	0.7(− 0.9:2.3)

Mean changes in GS (N/mm), PPT (kPa) and HPT (degrees Celsius) in groups randomized to SMT at the stiffest (‘stiff’) or most pain-sensitive (‘pain’) segment (columns). Subgrouped (rows) by responder status are defined as 0, 30, and 50% improvement thresholds in disability (ODI). Mean change refers to the difference in ODI between baseline and post-SMT, baseline, and follow-up, respectively, along with a 95% confidence interval. * = indicates a *p*-value < 0.05. *SMT* Spinal manipulative therapy, *GS* Global stiffness, *PPT* Pressure pain threshold, *HPT* Heat pain threshold, *ODI* Oswestry disability index

**Table 3:** Changes for responders (NRS) in lumbar stiffness and pain sensitivity (mechanical and thermal) following SMT

Time	Status	Threshold	GS		PPT		HPT	
			Pain	Stiff	Pain	Stiff	Pain	Stiff
Post-SMT	Responder	50%	−0.13(− 0.79:0.53)	−0.09(− 0.80:0.61)	179 (62:296)*	100(−25:225)	0.3(− 1.7:2.3)	0.9(− 1.1:3.0)
		30%	−0.30(− 0.83:0.23)	−0.05(− 0.56:0.45)	151 (55:246)*	40(− 51:132)	−0.3(− 1.9:1.3)	0.9(− 0.6:2.4)
		0%	0.01(−0.30:0.32)	− 0.09(− 0.41:0.23)	113 (57:169)*	41(− 17:98)	0.1(− 0.9:1.0)	0.3(− 0.6:1.3)
	Non-Responder	50%	0.04(− 0.26:0.33)	0.05(− 0.25:0.35)	83 (31:135)*	21(− 32:74)	0.0(− 0.9:0.9)	0.2(− 0.7:1.1)
		30%	0.12(− 0.20:0.44)	0.06(− 0.28:0.39)	81 (25:138)*	30(− 30:90)	0.1(− 0.8:1.1)	0.0(− 1.0:1.0)
		0%	0.03(− 0.52:0.59)	0.40(− 0.16:0.97)	52(− 48:153)	9(− 94:112)	0.0(− 1.7:1.7)	0.3(− 1.5:2.0)
Follow-up	Responder	50%	− 0.31(− 0.99:0.38)	0.22(− 0.48:0.92)	214 (91:336)*	91(− 34:217)	0.1(−2.0:2.1)	0.8(− 1.4:2.9)
		30%	− 0.24(− 0.80:0.32)	0.09(− 0.41:0.58)	182 (81:283)*	53(−36:142)	0.1(− 1.6:1.7)	1.1(− 0.4:2.7)
		0%	0.08(− 0.24:0.40)	− 0.01(− 0.35:0.32)	103 (46:160)*	69 (8:130)*	0.5(− 0.4:1.5)	0.9(− 0.1:1.9)
	Non-Responder	50%	0.15(− 0.16:0.45)	− 0.11(− 0.42:0.20)	66 (13:120)*	41(−15:96)	0.7(− 0.2:1.6)	0.8(− 0.2:1.7)
		30%	0.18(− 0.14:0.50)	− 0.12(− 0.47:0.23)	61 (4:118)*	46(− 18:109)	0.8(− 0.2:1.7)	0.6(− 0.5:1.6)
		0%	0.05(− 0.52:0.62)	−0.13(− 0.68:0.42)	45(− 59:149)	−5(− 105:95)	0.8(− 1.0:2.6)	0.5(−1.2:2.1)

Mean changes in GS (N/mm), PPT (kPa) and HPT (degrees Celsius) in groups randomized to SMT at the stiffest (‘stiff’) or most pain-sensitive (‘pain’) segment (columns). Subgrouped (rows) by responder status are defined as 0, 30, and 50% improvement thresholds in patient-reported low back pain (NRS). Mean change refers to the difference in NRS between baseline and post-SMT, baseline, and follow-up, respectively, along with a 95% confidence interval. * = indicates a *p*-value < 0.05. *SMT* Spinal manipulative therapy, *GS* Global stiffness, *PPT* Pressure pain threshold, *HPT* Heat pain threshold, *NRS* Patient-reported low back pain

#### Global stiffness

We observed no statistically significant changes within-group, nor were there any between-group differences, independent of responder status and randomization group. The minor differences observed appear to be spurious, with the largest differences seen in the subgroup with the lowest number of participants. A visual presentation can be found in Additional file 1.

#### Pressure pain threshold

Baseline PPT was not statistically significantly different between any of the responder thresholds.

Close examination of Tables 2 and 3 reveal that significant increases in PPT were observed in two contexts:

Pressure pain threshold increased for all 24 *responder* subgroups at both time points compared to baseline, independent of randomization groups (“pain” or “stiff”), clinical outcome measure (ODI and NRS), and responder threshold (0, 30, and 50%). This was statistically significant in 16 of the 24 comparisons. For the 24 *non-responder* subgroups, the findings were more discordant, and only statistically significant increases were observed in 9 of the 24 comparisons (8 for the *pain group*).

For the 24 *pain* subgroups, PPT again increased in all comparisons and statistically significant so in 21. The findings were also more discordant for the 24 *stiff* subgroups, and only statistically significant increases were observed in 5 instances (all *responders* subgroups).

**Fig. 2:**
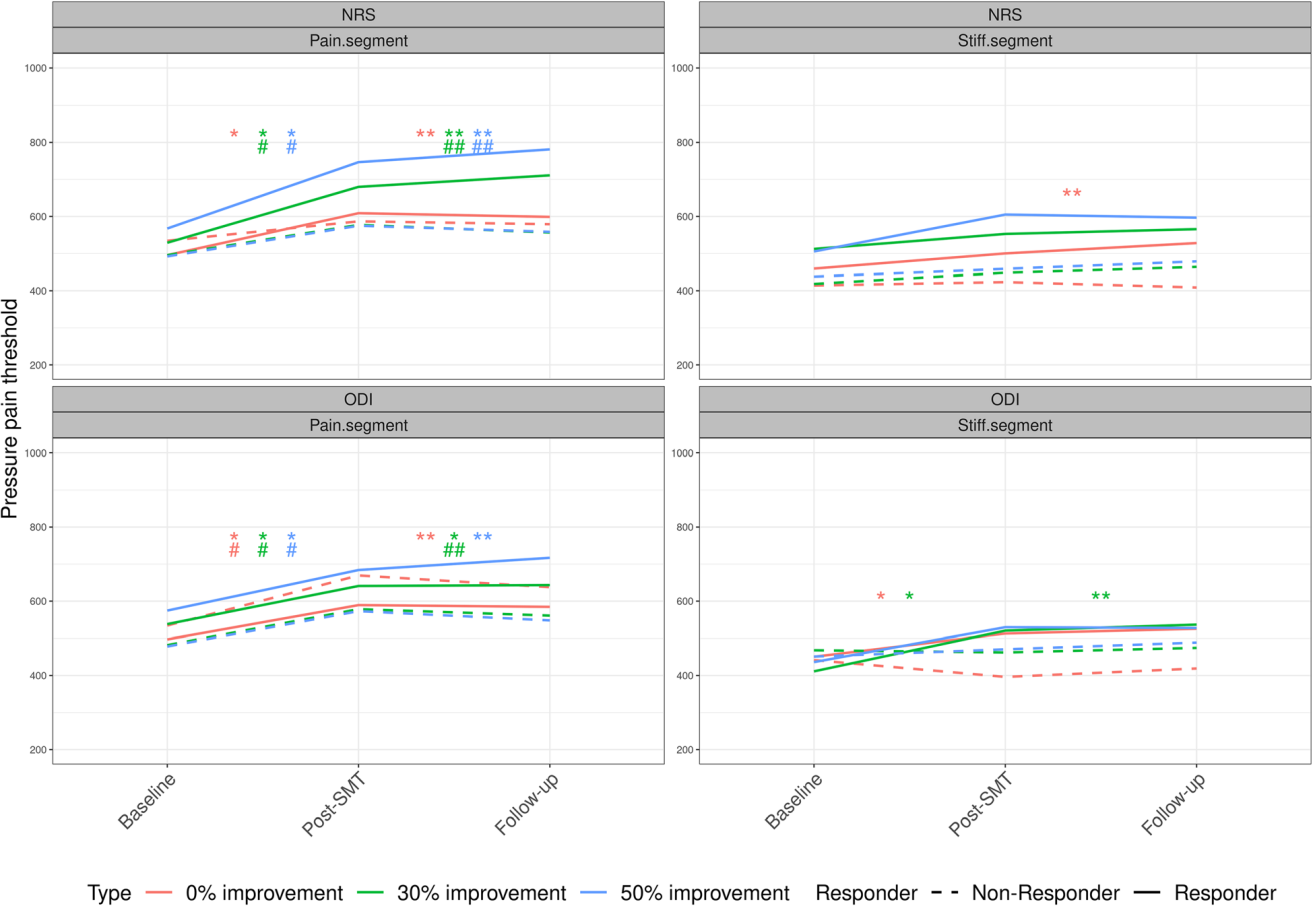
Changes in pressure pain threshold following SMT. Within-group mean changes in pressure pain threshold (kPa) for 50, 30, and 0% improvement in disability and patient-reported low back pain. Estimates are presented as means with 95% confidence intervals for each time-point and within-group significance level (*p* < 0.05) - presented as: * = Significant changes in responders from baseline to post-SMT. ** = Significant changes in responders from baseline to follow-up. # = Significant changes in non-responders from baseline to post-SMT. ## = Significant changes in non-responders from baseline to follow-up. SMT = Spinal manipulative therapy

In general, and for all responder thresholds, the change in PPT diminishes from post-SMT to follow-up. For further scrutiny of the between responder group differences, please see Table 4, and for ease of interpretation, the PPT changes are also presented visually in Fig. 2.

**Table 4:** Between-group changes in pressure pain threshold for responder vs non-responders following SMT

Time	Improvement	ODI		NRS	
		Pain	Stiff	Pain	Stiff
Baseline	50%	96(−70:262)	−42(− 232:148)	75(− 124:275)	13(− 195:222)
	30%	56(− 96:209)	−71(− 232:91)	33(− 136:203)	17(− 143:176)
	0%	−37(− 226:152)	− 84(− 275:108)	− 39(− 217:140)	−75(− 255:105)
Post-SMT	50%	15(− 92:122)	74(− 56:204)	96(− 32:225)	79(− 57:215)
	30%	6(−92:105)	115 (10:220)*	69(−41:180)	11(−98:119)
	0%	−43(− 172:86)	108(−4:219)	61(−54:176)	32(− 86:150)
Follow-up	50%	73(−39:184)	54(−73:181)	147 (14:281)*	51(−86:187)
	30%	26(−74:126)	119 (12:226)*	121 (5:237)*	8(− 101:117)
	0%	−16(−142:110)	98(−17:213)	59(−60:177)	74(−43:191)

Between-group mean differences (responder vs non-responder) in pressure pain threshold (kPa) at varying thresholds for improvement in disability and patient-reported low back pain (0, 30, 50%) in groups randomized to SMT at the stiffest (‘stiff’) or most pain sensitive (‘pain’) segment (columns). Estimates are presented as between-group differences, 95% confidence interval, and “*” indicates a *p*-value < 0.05. *ODI* Oswestry disability index, *NRS* patient-reported low back pain

#### Heat pain threshold

No statistically significant changes occurred for HPT. The data’s direction indicates minor improvements for the responders compared to non-responders independent of treatment allocation, but these were minor and not statistically significant. A visual presentation can be found in Additional file 1.

### Segmental proximity analysis

**Fig. 3:**
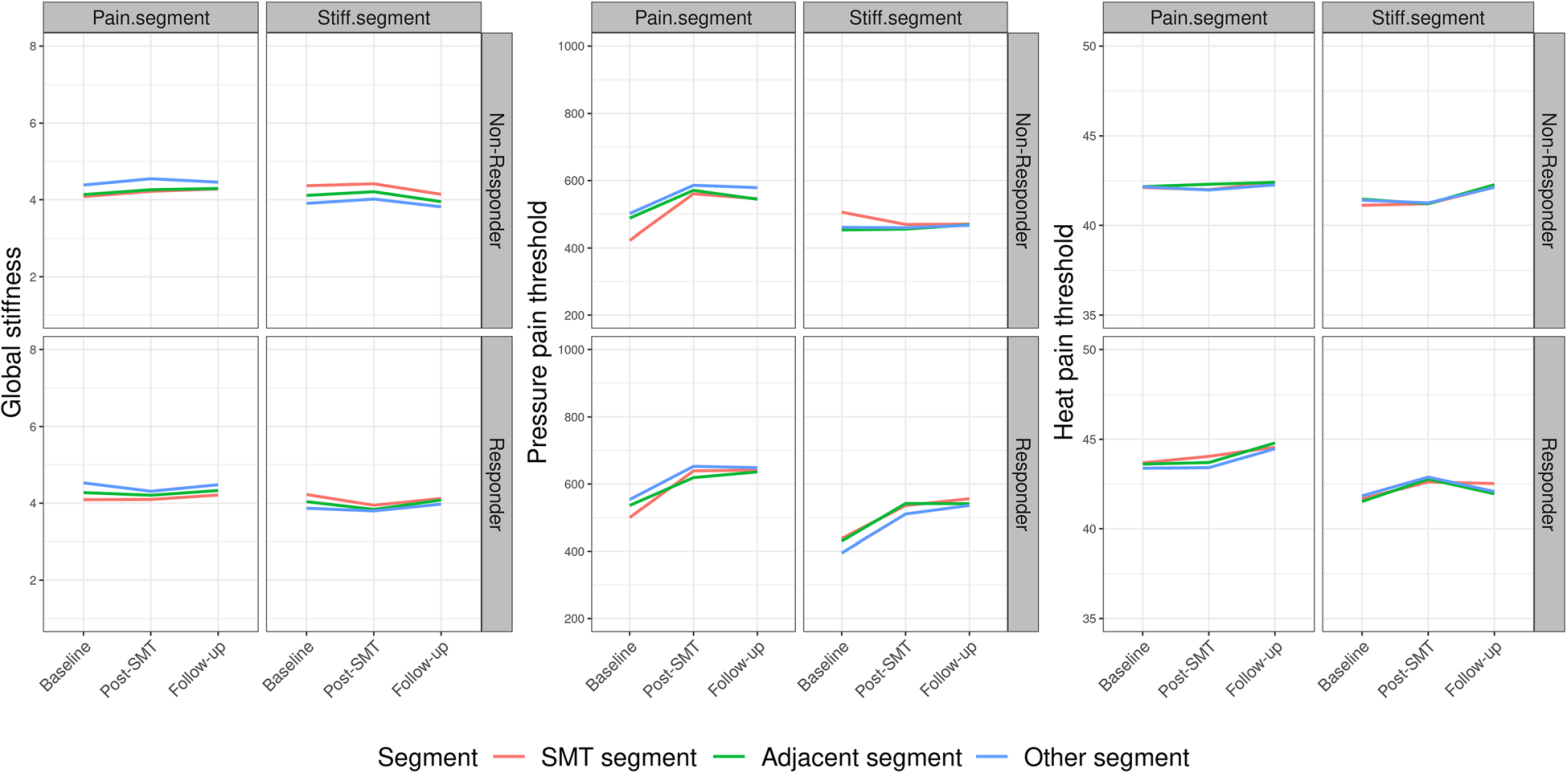
Segmental changes in pain sensitivity (mechanical and thermal) and lumbar stiffness following SMT. The segmental changes in global stiffness (N/mm), pressure pain threshold (kPa), and heat pain threshold (degrees Celsius) presented for 30% improvement in disability. Estimates are presented as mean and 95% confidence intervals for each time-point. Segments are divided into the segment targeted, the adjacent segments to the targeted segment, and all other segments. SMT = spinal manipulative therapy

When categorizing segments into i) target, ii) adjacent, and iii) other, no significant within-group differences were observed, and no discernible patterns emerged (see Figure f.seg). The same pattern is present for all outcomes (GS, PPT, HPT) for each segment and each responder threshold in ODI and NRS. For simplicity, we illustrate 30% ODI improvement in Fig. 3. The remaining figures are similar and can be found in Additional file 2.

## Discussion

The present analysis confirmed our previous results, namely that the segmental target impacted the increase of PPT, but added that responder status also had an isolated effect on PPT’s increase. The observations were consistent across multiple thresholds for the definition of responder status, suggesting the finding can be interpreted as robust. Only deep mechanical pain sensitivity was affected by responder status and the segment of the SMT target. Neither lumbar stiffness nor thermal pain sensitivity was affected. The PPT change appears to affect the whole lumbar spine as no obvious pattern emerged concerning the targeted, adjacent, and other segments.

This analysis presents new evidence regarding increases in PPT following SMT. More specifically, we observed an increase in two circumstances: i) when SMT was applied to the most pain-sensitive segment, in which case PPT increased irrespective of clinical response to treatment, and ii) in the clinical responder group, in which case PPT increased irrespective of where SMT was applied.

Multiple systematic reviews have indicated a non-specific decrease of mechanical pain sensitivity following SMT [26–29], and such an effect can explain the increase in PPT observed in the *pain segment group* irrespective of responder status (i). However, we did not observe such an effect in the *stiff segment group*. This may suggest that the analgesic effect may be greater when directed at pain-sensitive segments. In other words, this change in PPT may represent a localized, reflex-mediated reduction of pain sensitivity at a hyperalgesic segment.

Prior research also demonstrates that an experimental inhibition of a sensitized nociceptive trigger is followed by decreased experimental pain sensitivity [30, 31]. Our finding of increased PPT in the responder group independent of treatment allocation (ii) could be explained by such mechanism, i.e., a generalized decrease in experimental pain sensitivity following a successful reduction of clinical pain.

**Fig. 4:**
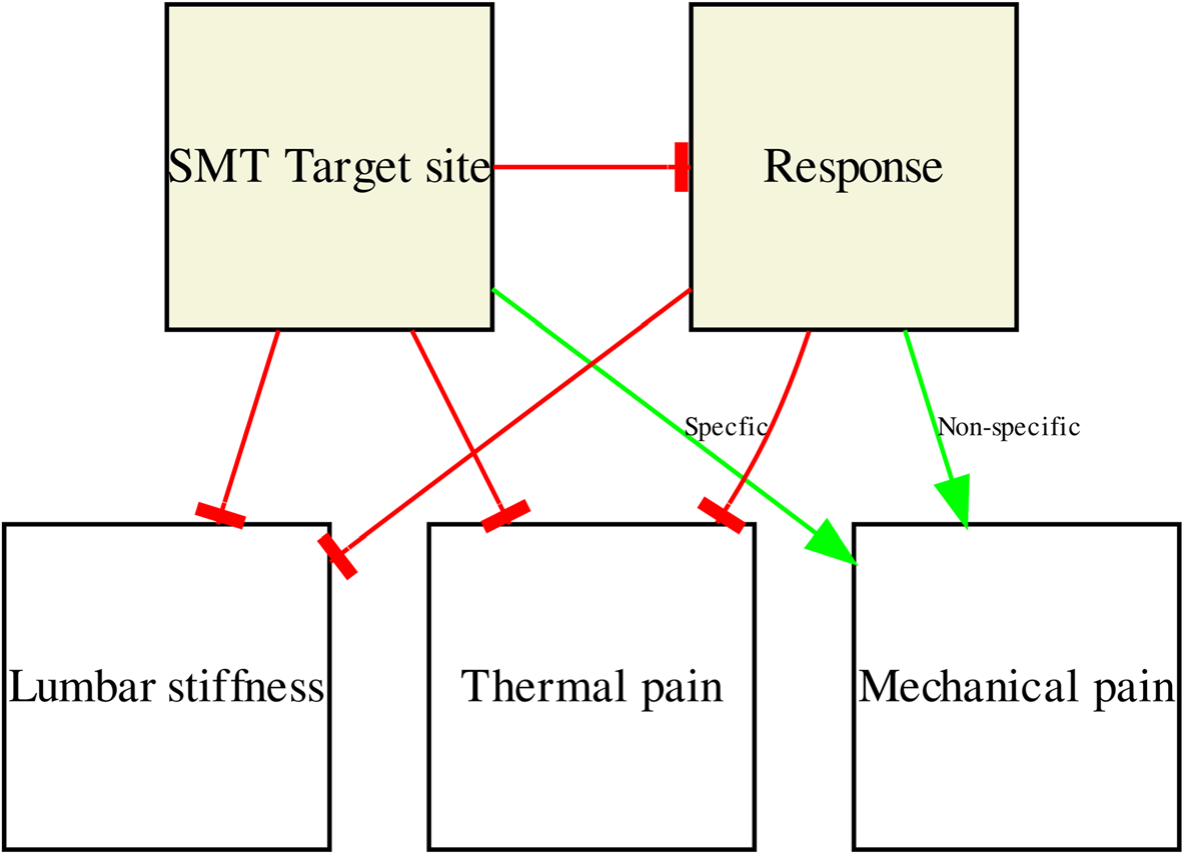
The pathway of experimental changes following SMT in chronic low back pain patients. A pathway of changes following SMT in lumbar stiffness and pain sensitivity (mechanical and thermal), both in general and dependent on the target site (*pain* segment or a *stiff* segment). A green arrow indicates a positive specific/non-specific effect on the outcome, and a red blocked arrow indicates no effect on the outcome. SMT = Spinal manipulative therapy

Therefore, the reduction of deep-tissue mechanical hyperalgesia in the present study could be explained by two parallel mechanisms: a *specific* neurophysiological effect of SMT on discrete hyperalgesic segments with no apparent clinical benefit, and a *non-specific*, general effect on pain sensitivity through successful treatment of a painful condition (Fig. 4).

It is apparent that the outcome changes in pain sensitivity following SMT were strictly limited to deep-tissue mechanical pain sensitivity. Heat pain threshold quantifies superficial (skin) pain sensitivity and if one assumes that LBP originates in the deep spinal tissues, it is conceivable that no effect on superficial pain sensitivity was observed in contrast to deep PPT.

Another RCT by Aspinall et al. compared SMT to sham and also examined whether being a “rapid responder” following SMT increased PPT at a higher rate than being a non-responder. The authors noticed small and non-significant increases in lumbar PPT immediately following SMT (up to 30 min) for the responders, but not so for non-responders [32]. The major differences between that and the current study were the intervention (1 vs. 4 sessions) and the follow-up time point (Immediately to 1 day vs. 2 to 4 weeks). While rapid responders may improve more clinically [33], it is questionable that clinically substantial changes in pain sensitivity would manifest so rapidly following a single treatment [3, 6]. Another observation by Aspinall et al. was that the responder group consisted of roughly equal numbers of actual and sham-SMT recipients [32], and there was no between-group difference in PPT increases following SMT or sham [34]. This raises questions about whether an increase in PPT following SMT results from the intervention, simple touch, or the passage of time. Finally, we performed the QST on a cohort of participants with chronic LBP, whereas the other study used volunteers with LBP [32]. Arguably chronicity presumes that this cohort had lower QST scores. This could potentially impact how PPT varies over time and whether it is of clinical relevance.

In recent decades, a range of QST procedures with well-delineated methodology (stimulus type, intensity, rate of application, quantification method etc.) has been used to publish data on pain sensitivity [35]. No such range of segmental spinal stiffness tests has been established [36], and it remains unclear which aspects of spinal stiffness are clinically relevant. Although the VT provides spinal stiffness measures, which are both standardized and objective, it is unknown to what extent these correlate to clinical procedures such as manual palpation [37]. It is entirely possible that other measures of segmental biomechanical function are more relevant than those obtained from the VT.

The current findings do not suggest that the experimental outcomes change only at the targeted segment, but instead, changes were observed for all of the lumbar segments. This contrasts the literature, particularly when SMT is applied in a highly controlled condition in animal models. Examining SMT in this fashion shows that the mechanistic outcomes are dependent on both the segmental target and the localized thrust on that segment [38, 39].

### Novel framework for SMT improvement

The underlying causes of low back pain are often obscure, and the connection to spinal stiffness is unclear. In degenerative joint and disc disease, spinal stiffness is likely affected, albeit pain is not always present. In other words, segmental spinal stiffness may be relevant to disability but potentially irrelevant to pain. Conversely, pain will tend to affect disability, irrespective of spinal stiffness.

Therefore, SMT may change pain perception through neurophysiological reflex mechanisms, thus changing both clinical pain and overall disability without actually improving segmental spinal stiffness. However, the difference in deep pressure pain sensitivity, which accompanies clinical improvement, may be unrelated to the SMT. Furthermore, SMT’s reflex-mediated segmental effect on deep pressure pain sensitivity, which does not translate into a clinical improvement, is probably of limited relevance.

### Methodological considerations

We observed a difference in the number of clinical responders when using disability compared to pain intensity. We consider the most likely reason for this difference to be that a reduction in pain intensity and improvement in disability does not necessarily correlate entirely [40]. Furthermore, the responsiveness of the two scales varies. The numerical rating scale is restricted to one domain with scores between 0 and 10, whereas ODI spans a range of 0 to 50 and records ten different domains. The smaller resolution of the 11-item NRS could limit the responsiveness, thereby translating into a smaller number of responders. However, this is speculative, and the authors are not aware of any data to support this.

Participants in the present study were LBP patients referred for assessment in a hospital setting. One of the formal criteria for such referral in Denmark is the insufficient effect of conservative management in the primary care setting. In other words, we must assume that LBP patients who respond favorably to SMT are underrepresented in this cohort compared to LBP patients in general [2]. The fact that around 23% of the participants experienced worsened disability after the intervention supports this assumption. This may further negatively impact the likelihood of positive clinical outcomes with SMT and affect the relationships between disability, pain, and experimental outcomes. This is speculative, however.

Another potential limitation is that we choose to dichotomize responder status at the final follow-up time point. We may miss some rapid responders directly following the fourth treatment to provide greater detail of the changes. However, the mean improvement at post-SMT and follow-up appears to be of equal size [3].

As stated in our previous paper [3], this was not a placebo-controlled study, and thus, the clinical improvement observed could be due to something other than the SMT. Therefore, the present findings do not speak to SMT’s clinical efficacy but rather to the underlying mechanism of any such effect. Furthermore, these results are limited to the cohort in question, as different possible outcomes could be observed in a primary care setting. Finally, the pressure algometry was applied by a double-headed probe. It is unknown whether this affects how the PPT scores change following SMT. Hence, caution should be taken when comparing our data to similar literature.

There are also strengths to consider; this was a relatively large cohort of chronic LBP patients seen in the secondary care sector. We measured the experimental outcomes both immediately following SMT and at 14-days follow-up and were able to correlate this with clinical improvements.

## Conclusion

Spinal manipulative therapy appears to have a segment-specific neurophysiological reflex effect that decreases deep mechanical pain sensitivity when directed at hyperalgesic segments, irrespective of clinical outcome. Furthermore, a generalized decrease in deep mechanical pain sensitivity was observed when clinical outcomes improve irrespective of the SMT target site. Stiffness and heat pain sensitivity were not found to respond in specific ways to SMT or based on clinical improvement.

## Supplementary Information

**Additional file 1.**

**Additional file 2.**

## Data Availability

Data is available upon reasonable request, please contact casper.nim@rsyd.dk

## Abbreviations

LBP: Low back pain
SMT: Spinal manipulative therapy
RCT: Randomized clinical trial
ODI: Oswestry disability index
NRS: Self-reported low back pain numerical rating scale
VT: VerteTrack
GS: Global stiffness
PPT: Pressure pain threshold
HPT: Heat pain threshold
