# Supplementary material for: Changes in pain sensitivity and spinal stiffness in relation to responder status following spinal manipulative therapy in chronic low Back pain: a secondary explorative analysis of a randomized trial
Source: BMC Musculoskelet Disord. 2021 Jan 6;22:23. doi: 10.1186/s12891-020-03873-3 (PMC7786943; doi:10.1186/s12891-020-03873-3)

## Additional file 1

Group mean differences in global stiffness for 50, 30 and 0% improvement in disability and patient reported low back pain. Estimates are presented as mean values for each time-point and within group significance level (p<0.05) - presented as: * = Significant changes in responders from baseline to post-SMT. ** = Significant changes in responders from baseline to follow-up. # = Significant changes in non-responders from baseline to post-SMT. ## =
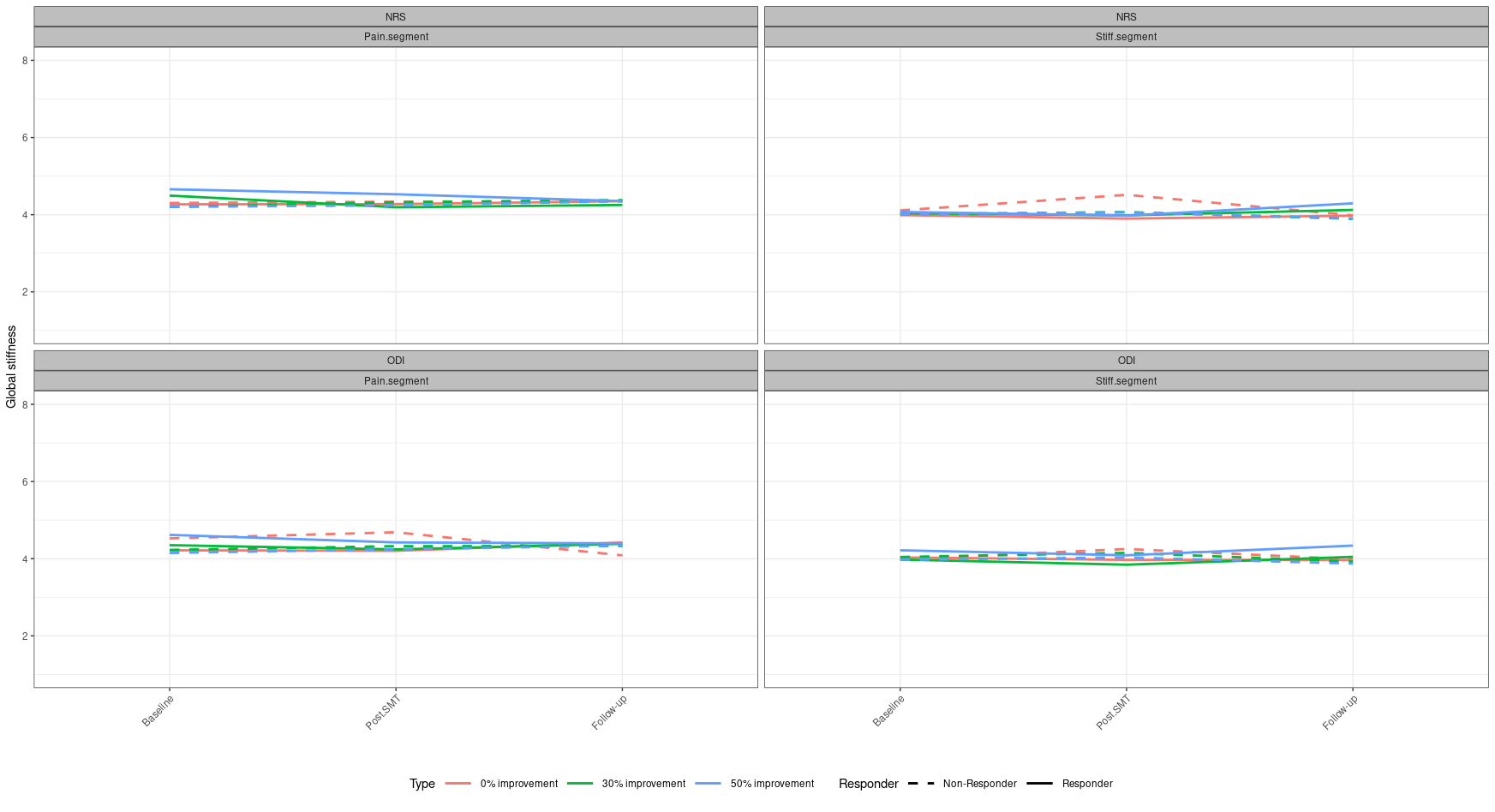
 Significant changes in non-responders from baseline to follow-up. SMT = Spinal manipulative therapy

Group mean differences in heat pain threshold for 50, 30 and 0% improvement in disability and patient reported low back pain. Estimates are presented as mean values for each time-point and within group significance level (p<0.05) - presented as: * = Significant changes in responders from baseline to post-SMT. ** = Significant changes in responders from baseline to follow-up. # = Significant changes in non-responders from baseline to post-SMT. ## = Significant changes in non-responders from baseline to follow-up. SMT = Spinal manipulative therapy


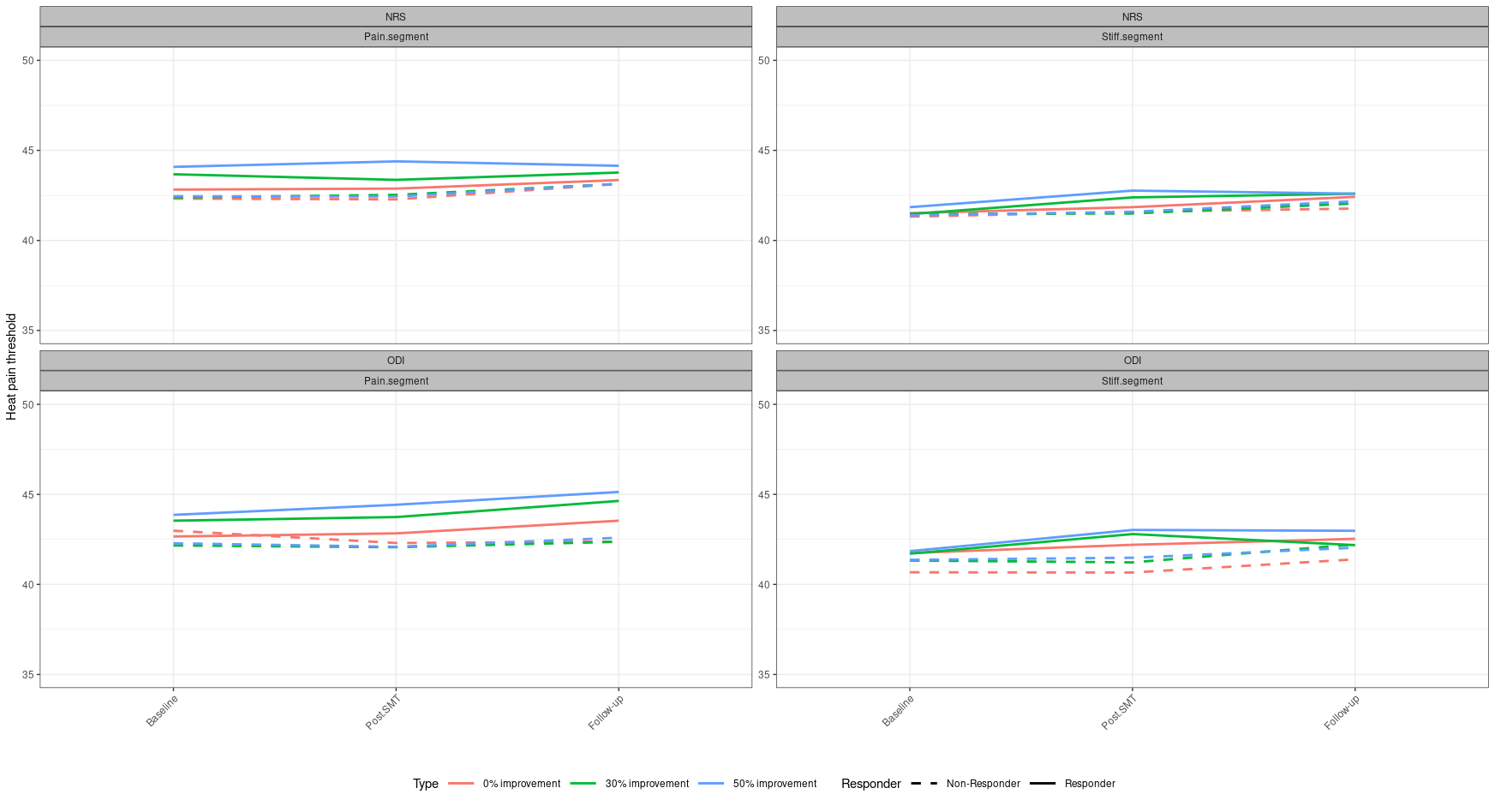

Supplement: Supplementary file 1 — Additional file 1. [file 12891_2020_3873_MOESM1_ESM.docx]
