# Supplementary material for: Changes in pain sensitivity and spinal stiffness in relation to responder status following spinal manipulative therapy in chronic low Back pain: a secondary explorative analysis of a randomized trial
Source: BMC Musculoskelet Disord. 2021 Jan 6;22:23. doi: 10.1186/s12891-020-03873-3 (PMC7786943; doi:10.1186/s12891-020-03873-3)

## Additional file 2

The segmental changes in pressure pain threshold, heat pain threshold, and global stiffness presented for 0% improvement in disability. Estimates are presented as mean and 95% confidence intervals for each time-point. Segments are divided into the segment targeted, the adjacent segments to the targeted segment and all other segments. SMT = spinal manipulative therapy


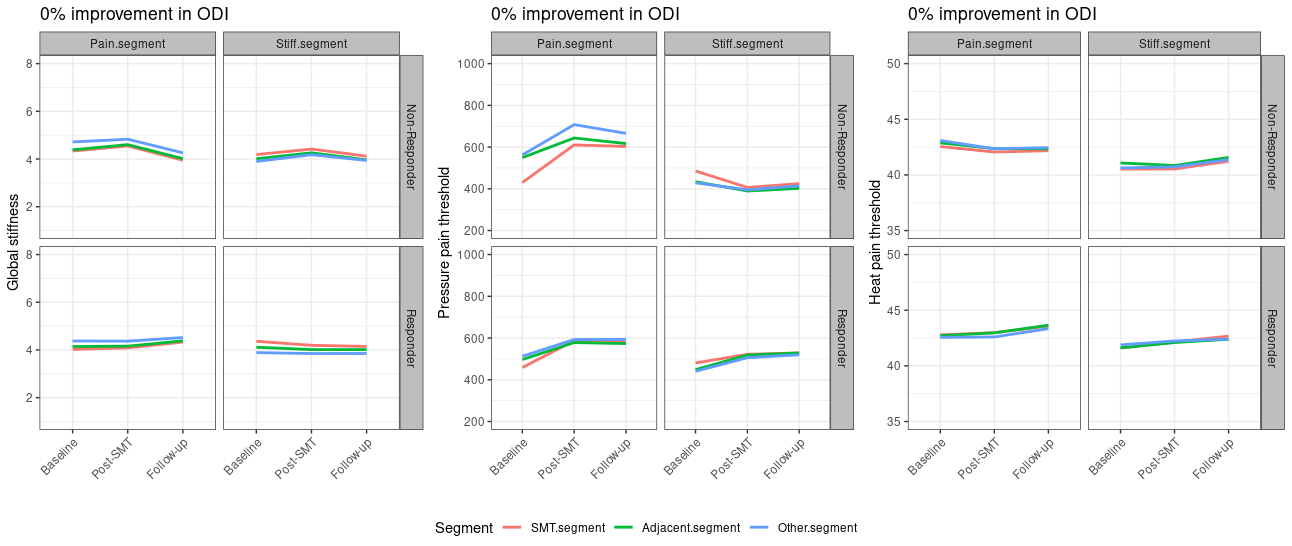


The segmental changes in pressure pain threshold, heat pain threshold, and global stiffness presented for 50% improvement in disability. Estimates are presented as mean and 95% confidence intervals for each time-point. Segments are divided into the segment targeted, the adjacent segments to the targeted segment and all other segments. SMT = spinal manipulative therapy


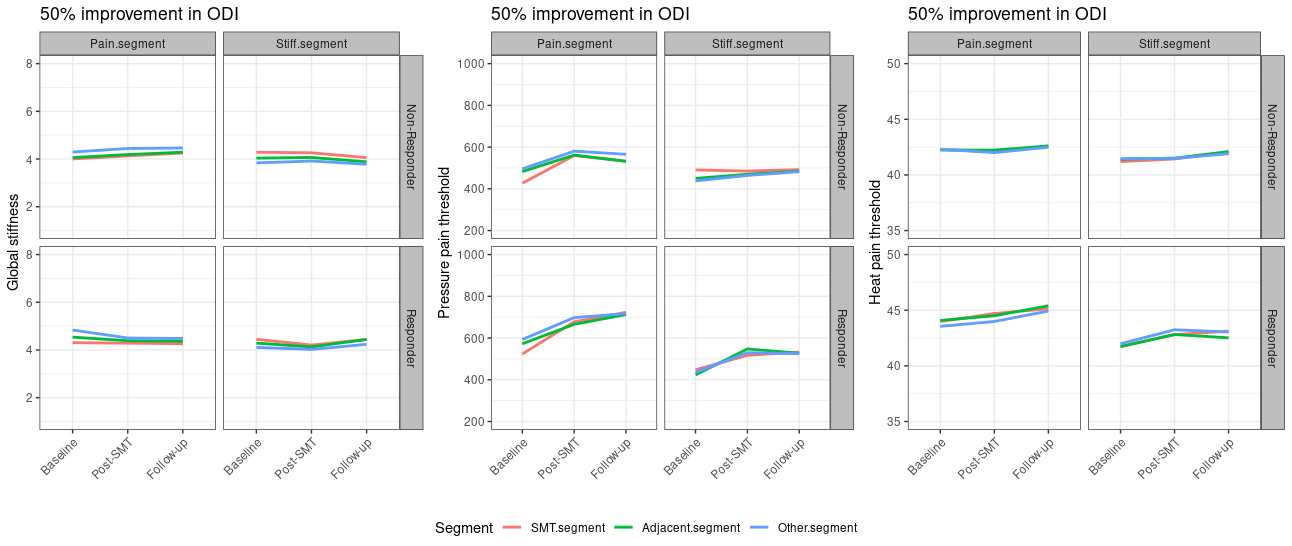


The segmental changes in pressure pain threshold, heat pain threshold, and global stiffness presented for 0% improvement in patient reported low back pain. Estimates are presented as mean and 95% confidence intervals for each time-point. Segments are divided into the segment targeted, the adjacent segments to the targeted segment and all other segments. SMT = spinal manipulative therapy


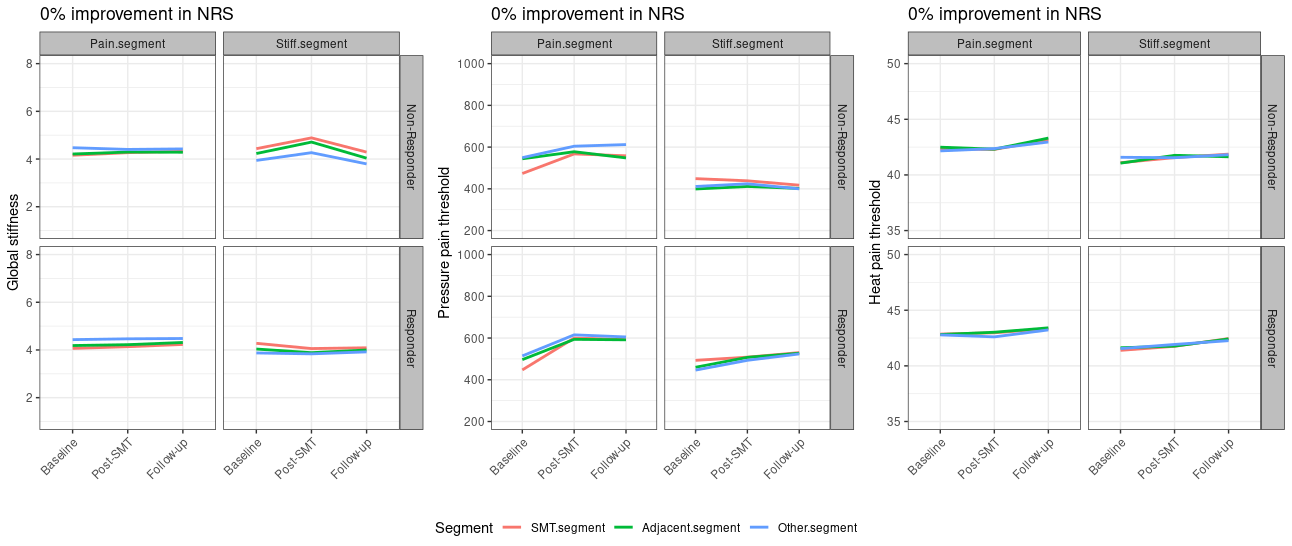


The segmental changes in pressure pain threshold, heat pain threshold, and global stiffness presented for 30% improvement in patient reported low back pain. Estimates are presented as mean and 95% confidence intervals for each time-point. Segments are divided into the segment targeted, the adjacent segments to the targeted segment and all other segments. SMT = spinal manipulative therapy


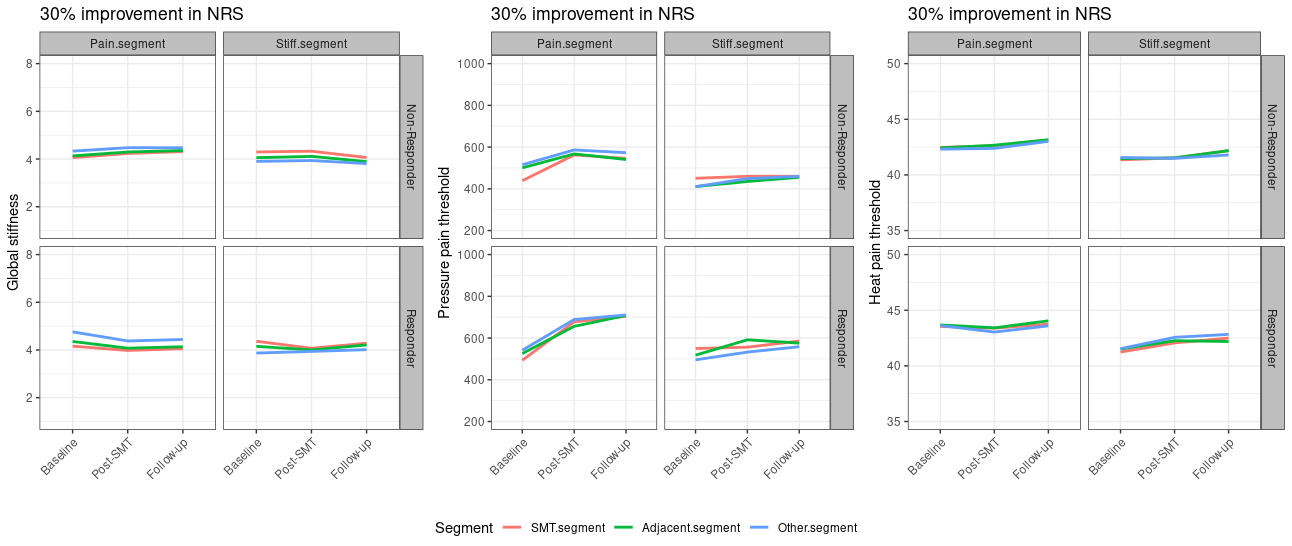


The segmental changes in pressure pain threshold, heat pain threshold, and global stiffness presented for 50% improvement in patient reported low back pain. Estimates are presented as mean and 95% confidence intervals for each time-point. Segments are divided into the segment targeted, the adjacent segments to the targeted segment and all other segments. SMT = spinal manipulative therapy


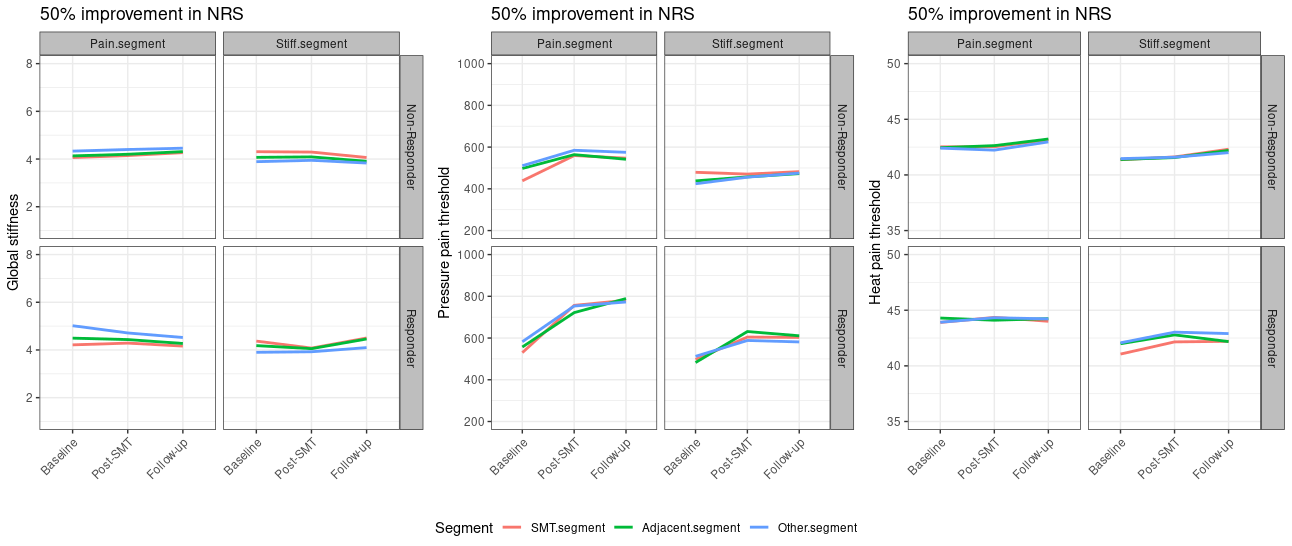

Supplement: Supplementary file 2 — Additional file 2. [file 12891_2020_3873_MOESM2_ESM.docx]
